# Novel and potential future therapeutic options in systemic autoimmune diseases

**DOI:** 10.3389/fimmu.2024.1249500

**Published:** 2024-03-15

**Authors:** Lili Balogh, Katalin Oláh, Soma Sánta, Nóra Majerhoffer, Tamás Németh

**Affiliations:** ^1^ Department of Physiology, Semmelweis University School of Medicine, Budapest, Hungary; ^2^ MTA-SE “Lendület” Translational Rheumatology Research Group, Hungarian Academy of Sciences and Semmelweis University, Budapest, Hungary; ^3^ Department of Rheumatology and Clinical Immunology, Semmelweis University, Budapest, Hungary; ^4^ Department of Internal Medicine and Oncology, Semmelweis University, Budapest, Hungary

**Keywords:** autoimmune disease, pathomechanism, treatment, inhibitor, antibody, clinical trial

## Abstract

Autoimmune inflammation is caused by the loss of tolerance to specific self-antigens and can result in organ-specific or systemic disorders. Systemic autoimmune diseases affect a significant portion of the population with an increasing rate of incidence, which means that is essential to have effective therapies to control these chronic disorders. Unfortunately, several patients with systemic autoimmune diseases do not respond at all or just partially respond to available conventional synthetic disease-modifying antirheumatic drugs and targeted therapies. However, during the past few years, some new medications have been approved and can be used in real-life clinical settings. Meanwhile, several new candidates appeared and can offer promising novel treatment options in the future. Here, we summarize the newly available medications and the most encouraging drug candidates in the treatment of systemic lupus erythematosus, rheumatoid arthritis, Sjögren’s disease, systemic sclerosis, systemic vasculitis, and autoimmune myositis.

## Introduction

Pathogenic autoimmunity occurs when specific self-molecules trigger a massive inflammatory response by autoreactive T or B cells and/or autoantibodies, which recruit innate and more adaptive immune cells. The consequent inflammation can be limited to a single organ (e.g., in autoimmune thyroiditis) or can affect several tissues in the body. These latter conditions, systemic autoimmune diseases, affect a significant proportion of the population. Their incidence is growing, they often appear as severe/organ- or life-threatening disorders, and a group of patients still cannot reach clinical remission.

**Figure 1:**
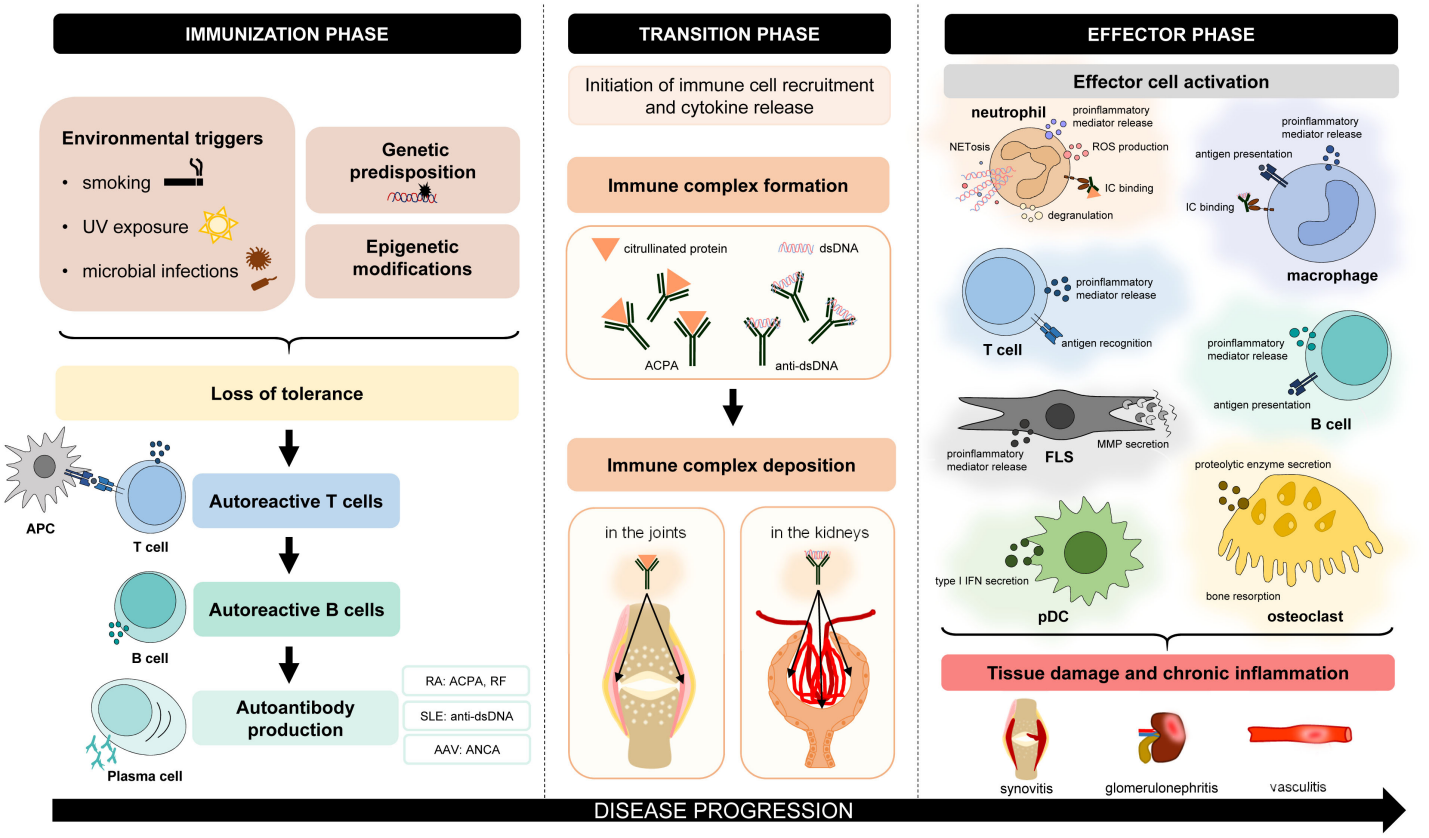
Common features of the pathogenesis of autoimmune disorders. The pathogenesis of different autoimmune diseases has many similar aspects. In general, genetic factors and environmental triggers collectively predispose to the development of autoimmunity. Due to the loss of immune tolerance, initiation phase leads to the appearance of autoreactive T lymphocytes, which contribute to the maturation of autoreactive B lymphocytes and autoantibody production. During the transition phase, complex immunological processes occur, such as immune complex formation and deposition or the initiation of immune cell recruitment. In the effector phase, different cell types are activated, which mediate tissue damage and chronic inflammation. AAV, ANCA-associated vasculitis; ACPA, anti-citrullinated protein antibody; ANCA, antineutrophil cytoplasmic antibody; APC, antigen-presenting cell; FLS, fibroblast-like synoviocyte; IC, immune complex; MMP, matrix metalloproteinase; NETosis, neutrophil extracellular trap formation; pDC, plasmacytoid dendritic cell; RA, rheumatoid arthritis; RF, rheumatoid factor; ROS, reactive oxygen species; SLE, systemic lupus erythematosus.

Autoimmune disorders are characterized by chronic inflammation, which can affect and damage various organs and tissues. Despite many differences in the pathogenesis and clinical manifestations of autoimmune diseases, there are also many similarities. Autoimmune disorders are believed to have three phases (**Figure 1**). In the first so-called “immunization” phase, patients are typically unaware of clinical symptoms (1). Often genetic factors (modified alleles) and environmental triggers (e.g., smoking, UV light, and microbial infections) collectively predispose to the development of autoimmunity (1). This process leads to the loss of immune tolerance to self-antigens by the appearance of autoreactive T lymphocytes, which escape central tolerance (2). As a consequence, self-reactive T lymphocytes can contribute to autoreactive B-lymphocyte development and autoantibody production (2). During the transition phase, complex immunological processes occur, such as immune complex formation or deposition (e.g., in the joints or the kidney) and the initiation of immune cell recruitment (3, 4). As a result, in the effector phase, many cell types become activated and initiate cellular responses. These effector functions mediate host tissue damage and maintain chronic inflammation. Based on these similar steps of pathogenesis, drug candidates targeting common participating factors may have beneficial effects in the treatment of various autoimmune disorders.

**Figure 2:**
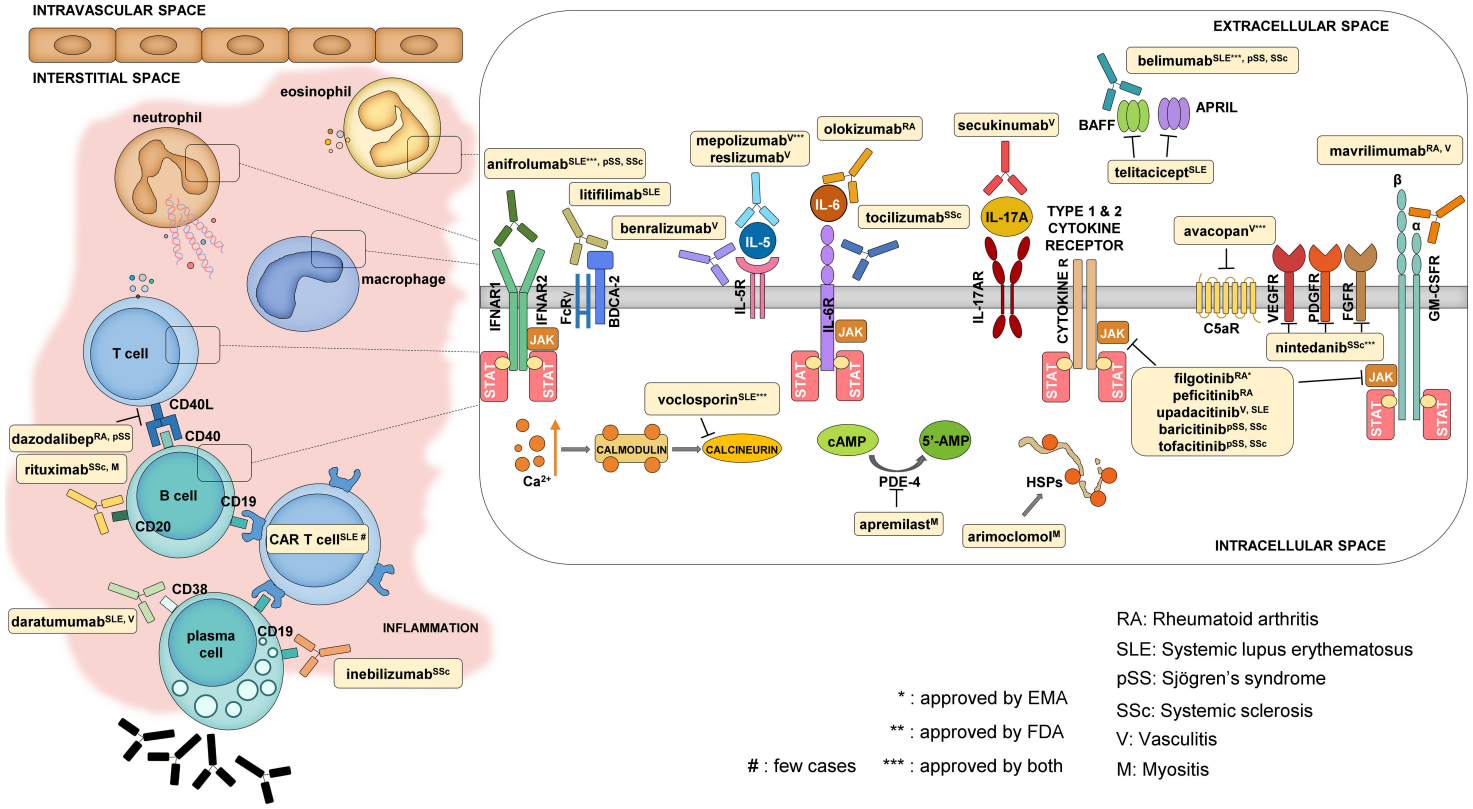
Mechanism of action of some selected newly approved therapies and promising drug candidates. The main cell types that contribute to the development of systemic autoimmune diseases are T and B cells, macrophages, neutrophils, and eosinophils. The reviewed drugs mostly have an impact on these cells either by targeting cellular functions from the extracellular side (like cytokine and cytokine receptor blockers) or by modifying intracellular signaling.

Patients living with systemic autoimmune diseases require lifelong therapy, and therefore, the choice of the right medication is key to the long-term outcome. Although several therapies are available, the desired complete remission is not achieved in a significant proportion of patients. However, the range of available therapies is constantly expanding due to the large number of drugs successfully tested in clinical trials. Here, we collected the most recently approved and the majority of the most promising forthcoming drugs to have an overview of this expanding field (**Table 1** and **Figure 2**).

**Table 1:** Newly approved drugs and some selected promising candidates from phase 2 or 3 trials.

Drug	Target	Status (approved or clinical study phase)	Disease	Refs.
Anifrolumab	Type I interferon receptor subunit 1	Approved (FDA, EMA) Phase 2 Phase 3	SLE Sjögren’s syndrome SSc	(5) NCT05383677 NCT05925803
Voclosporin	Calcineurin	Approved (FDA, EMA)	SLE	(6)
Belimumab	BAFF/BLyS	Approved (FDA, EMA) Phase 2 Phase 2	SLE SSc Sjögren’s syndrome	(7, 8) NCT01670565, NCT03844061 (9, 10)
Sifalimumab	Interferon-α	Phase 2	SLE	(11, 12)
Obinutuzumab	CD20	Phase 3	SLE	NCT04963296
Telitacicept	BAFF/BLyS and APRIL	Phase 3	SLE	(13, 14)
Atacicept		Phase 2	SLE	(15)
Ianalumab	BAFF receptor	Phase 3	SLE Sjögren’s syndrome	(16), NCT05639114, NCT05349214
Daratumumab	CD38	Phase 2	SLE	(17, 18) NCT04810754
Litifilimab	BDCA-2	Phase 3	SLE, CLE	(19, 20)
Low-dose IL-2	regulatory T cells	Phase 2	SLE	(21, 22)
Filgotinib	JAK1	Approved (EMA)	RA	(23–25)
Upadacitinib		Phase 2 Phase 3	SLE Takayasu’s arteritis, GCA	NCT03978520 NCT04161898, NCT03725202
Peficitinib	All JAKs	Approved (South Korea, Japan)	RA	(26, 27)
Mavrilimumab	GM-CSFR	Phase 2	RA, GCA	(28, 29)
Dazodalibep	CD40L	Phase 2 Phase 3	RA Sjögren’s syndrome	(30–32), NCT06104124
Olokizumab	IL-6	Phase 3	RA	(33–35)
Baricitinib	JAK1 and JAK2	Phase 2 Phase 4	Sjögren’s syndrome SSc	NCT05016297 NCT05300932
Nintedanib	VEGFR, PDGFR, FGFR	Approved (FDA, EMA)	SSc	(36)
Tocilizumab	IL-6R	Phase 3	SSc	(37)
Brodalumab	IL-17A receptor	Phase 3	SSc	(38)
Tofacitinib	JAK1 and JAK3	Phase 2	Sjögren’s syndrome SSc	NCT04496960 (39)
Inebilizumab	CD19	Phase 3	SSc	NCT05198557
Secukinumab	IL-17A	Phase 2	GCA	(40)
Avacopan	C5a receptor	Approved (FDA, EMA)	GPA, MPA	(41, 42)
Mepolizumab	IL-5	Approved (FDA, EMA)	EGPA	(43–45)
Reslizumab		Phase 2	EGPA	(46)
Benralizumab	IL-5R	Phase 3	EGPA	(47) NCT04157348
IVIG	?	Phase 3	DM	(48)
Apremilast	PDE-4	Phase 2	DM	(49)
Arimoclomol	HSP	Phase 3	IBM	(50) NCT04049097

DM, dermatomyositis; EGPA, eosinophilic granulomatosis with polyangiitis; EMA, European Medicines Agency; FDA, Food and Drug Administration; GCA, giant cell arteritis; GPA, granulomatosis with polyangiitis; HSP, heat shock protein; IBM, inclusion body myositis; IVIG, intravenous immunoglobulin; MPA, microscopic polyangiitis; RA, rheumatoid arthritis; SLE, systemic lupus erythematosus; SSc, systemic sclerosis.

## Novel therapies in SLE

### Recently approved therapies

Systemic lupus erythematosus (SLE) can affect multiple organs, most often in women of childbearing age, leading to a significant decrease in quality of life in the majority of the cases (4). The innate and adaptive immune systems are jointly responsible for the development of SLE (4). The presence of nucleic acid-containing immune complexes triggers type 1 interferon (IFN) production in plasmacytoid dendritic cells through a Toll-like receptor-dependent or receptor-independent pathway and activates several immune cells (4). The helper T cell population contributes to excessive autoreactive B cell activation and proliferation partly through CD40L–CD40 ligation, while decreased interleukin-2 (IL-2) production and regulatory T cell dysfunction are also seen in SLE (4). The presence of proliferation- and differentiation-promoting factors (e.g., BAFF/BLyS) helps the survival of self-reactive B cells, which can differentiate into autoantibody-producing plasma cells (4). During the last few years, novel therapies have been approved for the treatment of systemic lupus erythematosus.

While it has been known for several decades that type I interferons have an important role in the pathogenesis of SLE, it took a long time to have the first approved medication targeting this pathway. Anifrolumab is a monoclonal antibody against the type I interferon receptor subunit 1 (IFNAR1), which has been shown to be effective in a phase 3 clinical trial in SLE patients who received 300 mg intravenous injection every 4 weeks for 48 weeks according to the British Isles Lupus Assessment Group (BILAG)-based Composite Lupus Assessment (BICLA) response (5). Anifrolumab seemed to be a relatively safe biological therapy and was associated with a moderate increase in the incidence of herpes zoster compared to placebo (5). These data led to the approval of the drug by the US Food and Drug Administration (FDA) and the European Medicines Agency (EMA) of the European Union for non-renal manifestations. A 3-year long-term extension study — where the occurrences of non-opportunistic infections, malignancy, or major acute cardiovascular events were comparable with those of the placebo group — further proved a good benefit–risk ratio in patients with moderate-to-severe disease activity (51).

Voclosporin, which belongs to the calcineurin inhibitor family and mainly targets T cells, was found to be an effective therapy in Class III, IV, or V lupus nephritis patients at a 23.7-mg twice-daily dose in combination with background mycophenolate mofetil (MMF; 1 g/day) and low-dose corticosteroid (loCS) therapy compared to placebo with MMF plus loCS (in other words, voclosporin caused a superior complete renal response rate by week 52 in treated patients) (6). Meanwhile, voclosporin showed a good safety profile: the incidence of serious adverse events did not differ in the two groups (6). These findings led to the approval of the drug for the treatment of lupus nephritis by the FDA and EMA. Compared to tacrolimus, voclosporin shows predictable pharmacokinetics and does not need drug monitoring (52). Moreover, voclosporin therapy has a less severe impact on the lipid or electrolyte profile and has no effect on mycophenolate mofetil levels (52). Voclosporin could be also used in combination, which is considered to be a promising feature (53).

B cells are key players in the pathogenesis of SLE, and elevated serum levels of BAFF/BLyS can be detected in these patients (54). The anti-BAFF/BLyS monoclonal antibody belimumab was the first approved biological therapy for autoantibody-positive SLE patients; however, the indication was restricted to non-renal manifestations. In a 2-year, multicenter, randomized, double-blind, placebo-controlled, phase 3 trial, belimumab (at a 10-mg/kg monthly dose) plus standard therapy (mycophenolate mofetil or cyclophosphamide) had better renal response rates than standard therapy alone in lupus nephritis patients (7). The efficacy was stable, and no new safety concerns were raised during a 28-week, open-label extension study (8).

### Promising new drug candidates

The effect of anifrolumab is being tested in lupus nephritis patients. In a phase 2 study, the primary endpoint (change in baseline 24-hour urine protein–creatinine ratio at week 52) was not reached; however, more patients achieved complete renal response (CRR) with ≤0.5 mg/mg urine protein–creatinine ratio or sustained oral glucocorticoid dosage reduction (≤7.5 mg/day, from week 24 to week 52) in the intensified regimen group (receiving 900 mg anifrolumab three times and 300 mg after) compared to the placebo (all participants received mycophenolate mofetil and oral glucocorticoids) (55). Based on these results, the authors decided to proceed with a phase 3 clinical trial (ClinicalTrials.gov Identifier: NCT05138133). It is important to mention that there are other interferon-blocking agents in the pipeline. Sifalimumab is a fully human IgG1 κ monoclonal antibody that binds to the majority of IFN-α subtypes (11). In a randomized, double-blind, placebo-controlled study with moderate-to-severe active SLE patients, the 1,200 mg/month sifalimumab dose caused a significantly higher SLE responder index-4 (SRI-4) responder rate by week 52 compared to placebo, while the Cutaneous Lupus Erythematosus Disease Area and Severity Index (CLASI) responder rate also tended to be higher in the sifalimumab groups (11). Meanwhile, sifalimumab showed a tolerable safety profile, with a comparable serious adverse event rate with placebo, while herpes zoster infections were more frequent in sifalimumab-treated individuals (11). The tolerability was further strengthened by a more recent phase 2 study with Japanese patients (12). However, more investigations are needed to explore the efficacy of sifalimumab in active SLE patients.

In addition to belimumab, other B cell-targeted therapies have been also tried in SLE patients, and some have promising results. While rituximab, a chimeric anti-CD20 antibody, failed to show significant efficacy in a randomized, double-blind, phase 2/3 trial in patients with extrarenal SLE, this was probably due to an unfortunate study design, as there are many non-trial-based clinical data showing the successful off-label use of rituximab in individuals with SLE (56–58). Based on the positive clinical experiences with the (off-label) administration of rituximab under non-study-related clinical conditions, the monoclonal antibody was included in the 2019 update of the European Alliance of Associations for Rheumatology (EULAR) SLE treatment recommendation as second-line therapy for patients with refractory, severe non-renal SLE (Rituximab can also be found in the 2023 update with both renal and non-renal indications (59, 60). It is important to note that belimumab therapy following rituximab treatment could be beneficial (61). Ofatumumab, a fully humanized anti-CD20 monoclonal antibody, can be an alternative option for B cell depletion in patients who have previously had an allergic reaction to the off-label rituximab therapy (62). In a single-center retrospective case series of 16 patients with SLE, the use of ofatumumab was shown to be well-tolerated in 14 individuals, 12 patients achieved B cell depletion, and six lupus nephritis patients (out of 12) reached renal remission by 6 months (62). Ofatumumab seems to be a promising agent in the treatment of patients with life-threatening myeloid manifestations and in individuals with juvenile SLE according to case reports (63–65). Basic research data show that a new member of anti-CD20 antibodies, obinutuzumab, is more effective than rituximab in achieving B cell depletion (66, 67). In a phase 2 study, patients with lupus nephritis who received obinutuzumab with mycophenolate and corticosteroid therapy had a higher rate of achieving complete renal response at week 52 than the placebo group, while obinutuzumab was well tolerated (68). A phase 3 clinical trial is currently ongoing (ClinicalTrials.gov Identifier: NCT04963296).

Telitacicept is a fusion protein consisting of the transmembrane activator calcium modulator and cyclophilin ligand interactor (TACI) plus the Fc portion of human IgG (69). TACI can bind to both BAFF/BLyS and the proliferation-inducing ligand (APRIL) and was tested in moderate-to-severe Chinese SLE patients in a phase 3 clinical study (69). The subcutaneous administration of telitacicept caused an SLE responder index-4 (SRI-4) response in a significantly greater proportion of patients by week 52 than placebo (13, 14). Telitacicept also increased the serum C3 and C4 levels and reduced B cell numbers, serum IgM, and IgG titers while showing a good safety profile (14). Atacicept, a similar fusion protein, also seemed to be effective and well tolerated in a phase 2b clinical study (15). Ianalumab, a monoclonal antibody against the BAFF receptor, met the primary endpoint of SRI-4 response in a multicenter, randomized, double-blind trial in SLE patients (16). The chimeric antigen receptor (CAR) is a specially engineered synthetic protein, which is produced in a laboratory and consists of an antibody-derived antigen-binding, a hinge or spacer, a transmembrane, and two intracellular (the co-stimulatory and the CD3ζ) regions (70, 71). CAR-expressing T cells have the advantage of binding to the antigen in an MHC-independent manner (71). During CAR T cell therapy, T cells are collected from the peripheral blood of the patient, the CAR construct is inserted into the cells, and the CAR T cells are injected back into the patient (70). CAR T cell therapy caused a revolution in the treatment of refractory hematological malignancies, but its effect is also investigated in solid tumors (72, 73). In 2021, a German group published the case of a 20-year-old woman with severe and refractory SLE, having lupus nephritis, nephrotic syndrome, arthritis, pericarditis, pleurisy, and rash, where previous treatments with hydroxychloroquine, high-dose glucocorticoids, cyclophosphamide, mycophenolate mofetil, tacrolimus, rituximab, and belimumab were unable to control the disease and where anti-CD19 CAR T cell therapy resulted in complete remission (74). This observation was further strengthened by five more SLE patients with a refractory disease who received autologous anti-CD19 CAR T cell therapy and achieved drug-free remission (75). As a result of the treatment, all patients achieved low disease activity or remission by month 3, while their proteinuria and anti-dsDNA levels decreased and their C3 complement levels increased (75). Immune phenotyping of B cell before and after treatment revealed that the pathognomonic CD11c^+^CD21^lo^-activated memory B cells disappeared from the blood, while the therapy did not have a main effect on previous vaccination responses (75). Tolerability of the therapy was acceptable, as only one patient had a mild cytokine release syndrome that was successfully treated with tocilizumab (75). In addition to CAR T cell therapy, T cell engager agents also lead to B cell death *via* T cell activation; however, the data on this field are even more limited (preprint, doi: 10.31219/osf.io/fv7mn).

Daratumumab mainly targets plasma cells by binding to CD38 and is approved for the treatment of multiple myeloma (17). Ostendorf and colleagues described the cases of two patients with life-threatening refractory lupus who responded well to this anti-CD38 therapy and showed decreased urine protein–creatinine ratio, serum creatinine levels, serum anti-dsDNA titers, and SLEDAI-2K scores upon treatment (17). Moreover, daratumumab was successful in five out of six patients with refractory lupus nephritis in another case series investigation (18). An open-label, phase 2 clinical trial to explore the effectiveness of daratumumab in refractory SLE patients is ongoing (ClinicalTrials.gov Identifier: NCT04810754). Plasma cells are more susceptible to proteasome inhibition than other cell types, and therefore, drugs with this mechanism of action have been developed for the treatment of antibody- and plasma cell-dependent diseases like SLE (76). These drugs include bortezomib, which was previously approved by the EMA and FDA for the treatment of multiple myeloma. In one case study, dexamethasone therapy was combined with bortezomib infusion in a patient who was diagnosed with multiple myeloma in addition to SLE (77). Over two cycles, both diseases went into remission (77). In a study, bortezomib therapy reduced plasma cell counts in both the peripheral blood and the bone marrow, while it reduced circulating anti-dsDNA levels and SLE disease activity (78). However, several patients discontinued bortezomib therapy due to adverse events (78). In three out of five patients diagnosed with lupus nephritis, the bortezomib–glucocorticoid combination caused complete remission, and the therapy reduced circulating antibody levels and improved renal function according to another publication (79). In another study, bortezomib therapy caused complete remission in one out of 12 patients with lupus nephritis and partial remission in 10 patients, while two patients had to stop therapy because of adverse events (80). Compared to placebo in a double-blind clinical trial, half of the patients in the bortezomib group discontinued therapy because of adverse events (81). While the reduction in the antibody titer was not observed, bortezomib appeared to be effective in terms of the SLE responding index (81). However, cautious use is necessary due to many adverse events (81).

A plasmacytoid dendritic cell-specific C-type lectin, BDCA-2, has been shown to be able to suppress interferon production upon ligation (82). Litifilimab, a novel anti-BDCA-2 antibody, has been shown to decrease the interferon secretion of plasmacytoid dendritic cells and could reduce the articular involvement in SLE patients compared to placebo in a multicenter, phase 2 trial (19). Furthermore, subcutaneous litifilimab was effective in lowering the area of the involved skin in cutaneous lupus erythematosus (CLE) in another phase 2 clinical study (20). Meanwhile, the adverse event rate was similar in the litifilimab and placebo groups, with a moderate tendency to increased susceptibility to viral infections in CLE patients (19, 20). These led to the continuation of testing this agent in phase 3 trials in both SLE and CLE individuals (ClinicalTrials.gov Identifiers: NCT05352919 and NCT05531565, respectively).

Ikaros and Aiolos are transcription factors involved in the pathogenesis of SLE, where Ikaros mediates type 1 interferon production of plasmacytoid dendritic cells and B lymphocytes, while Aiolos is involved in the differentiation of B cells (83). Iberdomide is a cereblon modulator, which leads to the ubiquitination and degradation of both Ikaros and Aiolos and reduced CD19- and CD20-positive B cell numbers and type 1 interferon gene expression in SLE patients, while increasing regulatory T cell counts and serum interleukin-2 levels, pointing toward a reshaping of the function of the immune system (83). In line with these findings, iberdomide at a 0.45 mg oral daily dose significantly increased the percentage of patients achieving an SRI-4 response at week 24 compared to the placebo group, especially in subjects with a high Aiolos or type 1 interferon gene expression signature in a phase 2 clinical study (84). Most of the adverse events were mild to moderate and involved urinary or upper respiratory tract infections, while the serious adverse event rate was similar in the iberdomide and placebo groups (84).

Regulatory T cell dysfunction is a common feature in SLE, which can lead to immune dysregulation and the breakdown of immune tolerance (85). The recovery of regulatory T cell function by the administration of low-dose IL-2, which is an important regulator of Tregs, is another promising therapeutic agent in SLE (85). In a placebo-controlled pilot study, 30 patients received 1 million IU/day subcutaneous IL-2 every other day for 2 weeks, which was followed by a 2-week break, and this 4-week period was repeated twice (21). Despite of the fact that the low-dose IL-2 therapy did not reach its primary endpoint (the SRI-4 response rate at week 12), it showed a significant difference compared to the placebo in the response rate at week 24, while no serious adverse events occurred in the IL-2 group (21). Furthermore, the complete remission rate of low-dose IL-2-treated lupus nephritis patients was also significantly higher at both weeks 12 and 24, while IL-2-treated individuals had reduced 24-hour proteinuria in contrast to the placebo group (21). In line with this observation, serum albumin levels rose in those receiving low-dose IL-2 by weeks 12 and 24 (21). In a larger, multicenter, randomized, double-blind, placebo-controlled, phase 2 trial, 50 patients received subcutaneous IL-2 (1.5 million IU/day dose for 5 days, which was followed by weekly injections for 12 weeks) (22). While the primary endpoint (in the context of the SRI-4 response rate) was not reached in the total investigated population, a *post hoc* per-protocol analysis — which excluded patients from two sites, where the SRI-4 response rate was 100% in the placebo group — showed a statistically significant difference in connection with the primary endpoint, which encourages further investigations (22). The effect of efavaleukin α, an IL-2 mutein Fc fusion protein with a high affinity to CD25, is also intensively investigated in SLE: in a phase 1b trial, different doses of efavaleukin had a tolerable safety profile while causing a selective and prolonged regulatory T cell expansion (86).

There are some positive and promising experiences with the off-label use of intravenous immunoglobulin (IVIG) therapy in refractory SLE patients; however, controlled trials are needed to define its potential role in the treatment of SLE (87).

Deucravacitinib, the inhibitor of the Janus kinase TYK2 was tested in a phase 2 trial, where significantly more patients achieved the SRI-4 response at week 32 in the inhibitor-treated group compared to placebo (88). The JAK inhibitor upadacitinib and the BTK inhibitor elsubrutinib are also tested in SLE patients in a phase 2 clinical study (ClinicalTrials.gov Identifier: NCT03978520).

## What is new in the RA field?

Rheumatoid arthritis (RA) has a prevalence of 0.5%–1% in the population and can cause irreversible joint damage and loss of articular function in patients, while it is associated with higher cardiovascular morbidity and mortality (3, 89). Despite the fact that there is a growing number of available therapies, a significant proportion of patients still do not respond at all or adequately to treatment (difficult-to-treat RA) (90). Several cell types and mediators play important roles in the pathogenesis: resident cells in the synovium (e.g., macrophage-like synoviocytes and synovial fibroblasts) and recruited leukocytes (e.g., T and B cells and neutrophils), which become activated, leading to the release of proinflammatory cytokines, like tumor necrosis factor-alpha (TNF-α) and interleukin-6 (IL-6) (which became therapeutic targets in the everyday clinical routine) or granulocyte macrophage colony-stimulating factor (GM-CSF) (3). Different mediators signal through various tyrosine kinases [e.g., Janus kinases (JAKs)] and contribute to the development of joint damage, bone erosions, and the maintenance of chronic inflammation (3).

JAK inhibitors are important drugs for the treatment of RA. In addition to the three “older” agents (tofacitinib, baricitinib, and upadacitinib), filgotinib is a relatively new candidate. Filgotinib is mainly a JAK1 inhibitor, which interferes with the pathogenesis of rheumatoid arthritis by modulating the effects of proinflammatory cytokines (91). When tested as a monotherapy in a phase 3 clinical trial, patients receiving filgotinib had a significantly higher response rate compared to patients receiving placebo (23). It has also been shown that filgotinib (at both 100 and 200 mg/day dosages) was significantly more effective (in the context of the ACR20 response rate at week 24) in combination with methotrexate compared to methotrexate monotherapy (92). In a 52-week, placebo-controlled, phase 3 trial, 200 mg filgotinib was non-inferior to adalimumab therapy when the DAS28-CRP score was examined (both therapies were combined with methotrexate) (24). Filgotinib has been shown to be safe and effective both in combination with methotrexate or as monotherapy in a 4-year open-label extension study (25). As a consequence, filgotinib has been approved for moderate-to-severe RA by the EMA. In parallel with selectivity, the safety profile is expected to be more favorable than for non-selective JAK inhibitors (e.g., baricitinib); for instance, it has been suggested that filgotinib therapy is associated with a reduced risk of herpes zoster infection compared to other JAK inhibitors (93).

Another new JAK inhibitor, that has been studied in RA, is the pan-JAK inhibitor peficitinib (94). In two 52-week, placebo-controlled, phase 3 trials, peficitinib treatment as monotherapy (at both 100-mg or 150-mg once-daily dosages) or in combination with methotrexate resulted in a significant reduction in RA symptoms (26, 27). Peficitinib has been approved for the treatment of RA in South Korea and Japan.

Mavrilimumab is a fully human monoclonal antibody against the GM-CSF receptor α chain. GM-CSF plays a central role in the development of RA, and the binding of the cytokine to its receptor leads to the activation of the JAK/STAT signaling route (95). Compared to placebo, subcutaneous mavrilimumab monotherapy at 30-mg and 100-mg doses helped more patients to achieve a more than 1.2 decrease in the DAS28-CRP score by week 12 in a phase 2a clinical trial (96). Moreover, no major safety concerns were raised about its tolerability (28, 97).

CD40L–CD40 binding is required for the development of humoral immune response since it has a major role in B cell activation and the generation of plasma cells (98). Dazodalibep binds CD40L and inhibits the attachment of the ligand to its receptor. In a placebo-controlled, phase 2 trial, the authors found that the intravenous administration of dazodalibep led to a significant reduction in the DAS28-CRP score by day 113, while no major safety concerns were raised (30, 31).

Olokizumab is a humanized monoclonal antibody against the proinflammatory cytokine IL-6, which is involved in the pathogenesis of RA in various ways (99). Subcutaneous administration of olokizumab (64 mg every 2 weeks or 64 mg every 4 weeks in combination with methotrexate) resulted in significant improvements in symptoms and physical function in patients with inadequate response to methotrexate or TNF-α inhibitors, while olokizumab therapy was non-inferior to adalimumab considering the ACR20 response rate at 12 weeks in another trial (33–35). Meanwhile, a similar safety profile was observed as with other approved IL-6 blockers (33–35). One can speculate whether there are any major differences in the therapeutic features of blocking IL-6 rather than IL-6R. The efficacy and safety of olokizumab were compared to those of the two IL-6R blockers (tocilizumab and sarilumab) in a meta-analysis among methotrexate (MTX) non-responder RA patients (100). According to this, all three drugs showed very similar efficacy and safety (100).

The programmed cell death protein 1 (PD-1) on T cells has an important role in the downregulation of T cell activation upon its ligation to PD-L1. Peresolimab is a humanized anti-PD-1 monoclonal antibody that activates this inhibitory pathway (in other words, it has the opposite effects as checkpoint inhibitors) (101). In a phase 2a clinical trial, peresolimab treatment led to a significantly greater reduction in the DAS28-CRP score at week 12 compared to placebo, which raises the possibility to control autoimmune inflammation by influencing the PD-1 inhibitory pathway (101).

In summary, recently, two JAK inhibitors (filgotinib and peficitinib) have gained approval in the treatment of rheumatoid arthritis, while several monoclonal antibodies (altering the pathomechanism of RA from different directions) are the subject of promising research in the RA field.

## New drug candidates in the treatment of Sjögren’s syndrome

Sjögren’s syndrome, with a prevalence of 0.01%–0.72%, is mainly associated with exocrine gland dysfunction and consequent dryness of the mucous membranes but can also affect several internal organs (e.g., the respiratory tract) over time (102). In primary Sjögren’s syndrome (pSS) epithelial cells express a variety of immune regulatory molecules, which — through increased cytokine and chemokine production (e.g., IL-7, IL-17, and IL-23) — induce abnormal T and B cell function and autoantibody production, participate in the activation of interferon signaling, and induce the accumulation and activation of various immune cells, triggering chronic inflammation and dysfunction of the exocrine glands (102). Several cytokines that are involved in the pathogenesis signal through the JAK/STAT route. Since pSS is a relatively frequent systemic autoimmune disease with symptoms that significantly reduce the quality of life and which has no licensed targeted therapies at the moment, developing new potential drugs is highly necessary.

Efgartigimod is an antibody fragment that binds neonatal Fc receptors, which are important in the recycling of IgG molecules, thus prolonging the half-life of the antibodies. Efgartigimod, which is already used in the treatment of myasthenia gravis patients, is investigated in a phase 2 clinical trial (ClinicalTrials.gov Identifier: NCT05817669).

A possible target in pSS therapy is the BAFF/BLyS pathway; thus, ianalumab, a monoclonal antibody against the BAFF receptor, is being tested in a phase 3 study (ClinicalTrials.gov Identifier: NCT05349214). Ianalumab was tested in a multicenter, phase 2b clinical trial in pSS patients (103). The findings were promising, and the reduction of EULAR Sjögren’s syndrome disease activity index score from baseline could be detected at all doses (103). It is important to note that higher doses were related to a greater reduction of the ESSDAI score (103). Administering 300 mg ianalumab significantly reduced the Physician Global Assessment (PGA) score, and an increased stimulated salivary flow (mL/min) could be seen (103). In addition, two open-label, phase 2 clinical trials have been fulfilled, where the BAFF/BLyS targeting belimumab as monotherapy seemed to be efficient for pSS patients (9, 10).

In a further study, the combination of belimumab and rituximab was tested in a phase 2 clinical trial in pSS patients (104). Patients were randomized into four groups: placebo, i.v. rituximab, s.c. belimumab, and i.v. rituximab combined with s.c. belimumab. The safety profile of the combined therapy was similar to the safety of monotherapies (104). Furthermore, a near-complete depletion of CD20^+^ B cells in minor salivary glands and a greater and more sustained depletion of peripheral CD19^+^ B cells were observed with belimumab combined with rituximab compared to monotherapies (104).

Another B cell-targeting therapy, telitacicept, has shown good efficacy and safety in a phase 2 clinical trial while reducing IgG, IgM, and IgA levels and CD19-positive B cell numbers (105). Telitacicept was administered subcutaneously every week in a 160-mg or 240-mg dose, and statistically significant improvements were found in the ESSDAI score in the 160 mg telitacicept group compared to the placebo group; however, there was no statistically significant difference between the 240-mg and the placebo groups (106).

Dazodalibep is a CD40 ligand antagonist that blocks the interaction between B cells and T cells. In a phase 2 clinical trial, dazodalibep was found to be tolerable and effective in patients with Sjögren’s syndrome (32). Therefore, recently, a phase 3 clinical trial was started, evaluating the efficacy and safety of dazodalibep in participants with Sjögren’s syndrome with moderate-to-severe systemic disease activity (ClinicalTrials.gov Identifier: NCT06104124). Iscalimab is an anti-CD40 monoclonal antibody. The effect of iscalimab was investigated in a multicenter, double-blind, placebo-controlled, phase 2 clinical trial, where patients received either subcutaneous iscalimab (at a 3 mg/kg dose) or intravenous iscalimab (at a 10 mg/kg dose) (107). The findings were promising, as the intravenous treatment resulted in a significant lowering of the ESSDAI score compared to placebo; however, there was no significant difference in regard to the ESSDAI score between subcutaneous iscalimab and the placebo group (107).

Type I interferons have important roles in the pathogenesis. Currently, there is an ongoing phase 2 clinical trial investigating the safety and efficacy of anifrolumab in pSS subjects (ClinicalTrials.gov Identifier: NCT05383677). 

Deucravacitinib, a TYK2 inhibitor, is already in use for treating plaque psoriasis in multiple countries and demonstrated positive outcomes during phase 2 clinical trials in patients with psoriatic arthritis and patients with SLE (88, 108, 109). Deucravacitinib could suppress IFN and B cell pathway markers in lupus patients; therefore, it could be useful in the treatment of patients with Sjögren’s syndrome, where IFN and B cell pathways have important roles in the pathogenesis (110). Accordingly, a phase 3 clinical trial is ongoing (ClinicalTrials.gov Identifier: NCT05946941).

The effect of other JAK inhibitors is intensively investigated. Baricitinib is tested in a phase 2 clinical trial that examines the efficacy of hydroxychloroquine (HCQ) and baricitinib combination therapy *versus* HCQ alone (ClinicalTrials.gov Identifier: NCT05016297). Tofacitinib is also in a phase 2 trial for pSS, which investigates the improvements in EULAR Sjögren’s Syndrome Patient Reported Index (ESSPRI) with a 5-mg twice-daily dose (ClinicalTrials.gov Identifier: NCT04496960).

## Systemic sclerosis in the focus

Systemic sclerosis is triggered by microvascular damage and immune activation, which leads to systemic fibrosis and can result in multi-organ failure, while the disease itself is really difficult to treat (111). The development of systemic sclerosis requires the activation of various immune (e.g., T or B cells, macrophages, and dendritic cells) and non-immune cell types (e.g., endothelial cells, fibroblasts, and smooth muscle cells) (111). Danger-associated molecular pattern (DAMP)- or immune complex-activated immune cells produce various cytokines, such as type I IFN or IL-6, which signal through the JAK/STAT pathway, and contribute to angiogenesis, wound healing, and fibrosis (111).

Interstitial lung disease (ILD) is a life-threatening complication of systemic sclerosis (SSc). Pirfenidone is an anti-inflammatory, anti-fibrotic drug inhibiting TGF-β and TNF-α. A phase 2 clinical trial failed to find any significant difference between the pirfenidone and placebo groups (112). However, 94.1% of the patients in the pirfenidone group showed stabilization or improvement in forced vital capacity (FVC), which led to a phase 3 trial (112). As a consequence of a successful phase 3 clinical trial that investigated the efficacy and safety of nintedanib, a small molecule tyrosine kinase inhibitor, the molecule is now a possible therapeutic option for SSc-associated lung fibrosis (36).

Previous studies showed that the classic proinflammatory cytokine IL-6 has a massive profibrotic effect; IL-6 levels are elevated in SSc patients and correlate with the thickness of the skin (113). In a phase 3 trial, the IL-6 receptor-blocking tocilizumab could preserve lung function in early SSc patients with ILD and elevated acute-phase reactants; however, it had a poor effect on skin fibrosis (37).

Skin fibrosis and pruritus cause a life-quality reduction in SSc patients; therefore, it is necessary to have more available therapeutic options. One of the cytokines that play important roles in the pathophysiology of SSc is IL-31, which is associated with pruritus (114). Nemolizumab (an IL-31 receptor inhibitor) is now in phase 2 clinical trial (ClinicalTrials.gov Identifier: NCT05214794). An encouraging multicenter, phase 3 trial has shown great efficacy of brodalumab, an anti-IL-17A-receptor monoclonal antibody on decreasing skin thickening (38). Brodalumab achieved a rapid, significant, and sustained reduction of the modified Rodnan skin score (mRSS) and inhibited the development of new digital ulcers (38). Brodalumab also demonstrated a positive effect on respiratory function and suppressed the progression of lung lesions (38). Abatacept, the inhibitor of the costimulatory molecules CD80 and CD86 on antigen-presenting cells, which, as a consequence, blocks T cell activation, was investigated in two separate double-blind, phase 2 trials and had positive outcomes (both clinical trials evaluated the change in mRSS) (115, 116). Despite the fact that the change in mRSS was not statistically significant, both studies found that abatacept was clinically effective, therefore suggesting the initiation of phase 3 clinical trials (115, 116). CD30^+^ lymphocytes are present in the skin biopsies from SSc patients, who tend to have increased serum CD30 levels (117). A chimeric anti-CD30 antibody, brentuximab, was tested in a phase 2b clinical trial, where the results were promising, as a significant decline of the mRSS score was detected (118). A further potential therapy for skin involvement could be the tyrosine kinase inhibitor imatinib according to a phase 2 trial, which showed significant mRSS score reduction, while the efficacy on lung involvement seemed to be poor (119).

Pulmonary arterial hypertension (PAH) is a severe consequence of SSc, which can result in heart failure. Ambrisentan, an endothelin receptor type A-selective antagonist, caused significant improvements in hemodynamic parameters like cardiac index or pulmonary vascular resistance. However, the mean pulmonary arterial pressure, the primary endpoint, only showed a tendency of improvement (120). In a multicenter, double-blind study, the efficacy of rituximab was investigated in SSc-ILD, where the 6-minute walk test showed a non-statistical improvement (121).

The JAK/STAT signaling pathway is significantly activated in SSc patients (122). The JAK inhibitor tofacitinib and baricitinib can cause improvement in lung function in ILD and mRSS score in skin fibrosis according to a systematic review of the literature (123). Tofacitinib showed trends of improvement during phase 1 and 2 clinical trials in clinical outcome measures (e.g., in mRSS) in patients with early diffuse cutaneous systemic sclerosis (39). In a phase 2 trial, baricitinib showed significant improvement in mRSS score and a favorable clinical effect on lung function and healing digital ulcers (124). Despite the fact that no information could be found on the results of a phase 3 trial, a phase 4 clinical trial is currently ongoing investigating the effects of baricitinib (ClinicalTrials.gov Identifier: NCT05300932). Itacitinib, another JAK/STAT pathway inhibitor, is being investigated in a phase 2 clinical trial (ClinicalTrials.gov Identifier: NCT04789850).

Since increased expression and activation of type-1 IFN-regulated genes have been reported in SSc patients, it might be beneficial to use treatments against these targets (125). Therefore, anifrolumab is undergoing a phase 3 clinical trial in scleroderma patients (ClinicalTrials.gov Identifier: NCT05925803).

Another promising therapeutic option in SSc patients is B cell inhibition. For instance, phase 2 clinical trials testing belimumab with or without rituximab (in combination with MMF) are currently ongoing (ClinicalTrials.gov Identifiers: NCT01670565 and NCT03844061). Moreover, the previously approved drug for neuromyelitis optica, inebilizumab (a CD19 inhibitor), is under phase 3 trial investigations for possible use in SSc (ClinicalTrials.gov Identifier: NCT05198557); however, there are no data on phase 2 results. During a phase 1 trial, inebilizumab therapy had a tendency to lower the mRSS score; however, it had no effect on FVC in ILD-associated SSc (126). Anti-CD19 CAR T cell therapy was used in a 60-year-old man with diffuse cutaneous systemic sclerosis who had lung and myocardial fibrosis, pulmonary arterial hypertension, Raynaud’s phenomenon, and arthritis (127). The therapy showed great efficacy: pulmonary fibrosis remained stable; right ventricular strain, arthritis, and skin fibrosis displayed a tendency of improvement; RNA polymerase III autoantibodies were no longer detectable (127).

## Controlling systemic vasculitis

### Large-vessel vasculitis

The large-vessel vasculitis group includes two systemic diseases, giant cell arteritis (GCA) and Takayasu’s arteritis, which have many similarities in the pathogenesis and can cause severe complications or death, while their treatment options are limited (128). During the initiation of the autoimmune inflammation, autoreactive T-helper cells reach the vessel wall, differentiate into cytokine-producing Th1 and Th17 cells, and recruit macrophages to the inflamed area (129). The inflammation is maintained by several mediators (e.g., by IL-23, IL-17, or GM-CSF) and signaling pathways (e.g., through the JAK/STAT signaling route) (129).

IL-17 inhibition could contribute to lowering the maintained dose of corticosteroids while decreasing the risk for relapse (129). Secukinumab is a monoclonal antibody targeting IL-17A. In a placebo-controlled, phase 2 study, Wenhoff and colleagues found that significantly more secukinumab-treated GCA patients were in remission at weeks 28 and 52 compared to the control group, while no unexpected safety signals were detected (40).

While IFNγ-secreting Th1 cells are quite resistant to corticosteroid therapy, they are dependent on the JAK/STAT signaling pathways, meaning that JAK1 and 3 inhibitors could be therapeutically beneficial (129). Unlike therapeutic monoclonal antibodies, these drugs can be orally administered, which can be more comfortable for the patients. In a case study involving five patients suffering from refractory Takayasu’s arteritis, four patients showed a reduction in symptoms, after tofacitinib therapy (5 mg twice daily), while two patients could have their basal glucocorticoid therapy reduced, and none of the five patients had adverse effects (130). Koster and colleagues treated 15 patients with baricitinib: 14 patients completed the 52-week treatment and during the investigated time period, only one patient had a relapse, and the other 13 patients could reach corticosteroid discontinuation and remained in remission (131). More patients with Takayasu's arteritis achieved remission after 12 months and remained in remission with 5-mg twice-daily tofacitinib compared to methotrexate in a prospective study (both therapies were combined with glucocorticoids) (132). Patients with Takayasu’s arteritis are being recruited for the clinical trial of upadacitinib, another JAK inhibitor (ClinicalTrials.gov Identifier: NCT04161898). There is also an ongoing clinical trial with upadacitinib for GCA (ClinicalTrials.gov Identifier: NCT03725202).

GM-CSF is believed to be one of the key factors in the pathogenesis of GCA (129). During a placebo-controlled, phase 2 clinical trial, 150 mg subcutaneous mavrilimumab combined with glucocorticoids performed better in terms of time to flare, compared to the placebo-glucocorticoid combination (29). Of patients who received mavrilimumab, 83% were in sustained remission at week 26, while only 50% were in remission in the placebo group (29).

Ustekinumab, a monoclonal antibody targeting IL-12 and IL-23, is currently being tested in a phase 2 study for the treatment of GCA (ClinicalTrials.gov Identifier: NCT03711448). There is also an ongoing phase 2 trial with the anti-IL-23 antibody guselkumab for GCA (ClinicalTrials.gov Identifier: NCT04633447).

### Small-vessel vasculitis

Antineutrophil cytoplasmic (auto)antibody (ANCA)-associated vasculitides (which belong to the small-vessel vasculitides) form a group of severe autoimmune conditions with possible involvement of nearly all kinds of tissues and sometimes result in severe organ (e.g., lung or kidney) damage, making clinical settings and therapeutic decisions difficult for patients and their doctors (133).

In the pathogenesis of ANCA-associated vasculitis, the adaptive immune system loses tolerance to neutrophil molecular components like myeloperoxidase or proteinase-3, resulting in the production of ANCAs, which trigger the activation of neutrophils (133). Other immune cells (e.g., B cells, monocytes, and macrophages) and humoral factors like C5a are also involved in mediating vessel wall inflammation (133).

It is no longer a question that alternative complement activation has a vital role in the pathogenesis of ANCA-associated vasculitis (AAV) (134). Avacopan is an orally available inhibitor of the C5a receptor 1 (135). Avacopan is a potential steroid-sparing drug: a 30-mg twice-daily dose with or without prednisolone background was non-inferior to glucocorticoid therapy, considering the proportion of patients achieving 50% reduction or more in the Birmingham Vasculitis Activity Score (BVAS) by week 12 (all patients received cyclophosphamide or rituximab) (41). In a phase 3 trial, at week 26, avacopan was as good as corticosteroids in maintaining remission, and at week 52, avacopan seemed to be the more effective (136). Avacopan was first approved for two forms of AAV in Japan and in the USA in 2021, which was followed by the EMA approval (42). 

Eosinophilic granulomatosis with polyangiitis (EGPA) is a rare AAV subtype, which is characterized by eosinophilic inflammation with bronchial asthma and small-vessel vasculitis (137). Th2-derived cytokines seem to play a vital role in the pathophysiology of the disease (137). IL-5 is critical for the proper maturation and activation of eosinophils, which are key players in the development of EGPA (138). Anti-IL-5 antibodies were originally used for treating severe asthma. In a multicenter, phase 3 clinical trial, 300 mg of subcutaneous mepolizumab led to a significantly increased duration of remission, and a larger proportion of patients remained in remission at weeks 36 and 48 compared to the placebo group (43). When combined with rituximab, a 100 mg every 4 week dose of mepolizumab-induction therapy could reduce asthma attacks while affecting sustained remission and having a steroid-sparing effect (45). (It is important to note that the licensed dose for EGPA is 300 mg every 4 weeks.) Reslizumab, another IL-5-blocking agent, was able to reduce the glucocorticoid dose in the therapy of EGPA patients, while it had a promising impact on outcomes of recipients (46). Benralizumab, an anti-IL-5 receptor antibody, which reduces eosinophil and basophil numbers, was found to be beneficial for preventing acute asthmatic flares in EGPA, and it could also contribute to steroid sparing (47, 139).

A very recent study was conducted with tofacitinib in granulomatosis with polyangiitis (GPA) with a small number of patients, and although this amount of patients was not enough for true safety assessment, the results are promising (140).

Recently, some promising case reports were published about the effect of daratumumab in patients with refractory ANCA-associated vasculitis (141, 142). Daratumumab treatment significantly reduced ANCA levels and caused fast and successful clinical improvements (141, 142). These promising results should be confirmed by clinical trials in the future.

## Potential future therapeutic options of autoimmune myositis

The group of idiopathic inflammatory myopathies consists of heterogeneous disorders, such as polymyositis, dermatomyositis, antisynthetase syndrome, immune-mediated necrotizing myopathy, inclusion body myositis, and overlap myositis syndromes (143). These chronic diseases are mediated by various humoral and cellular factors such as T cells and myositis-specific autoantibodies (143). A frequent common symptom is muscle weakness and muscle pain, but there can be several other manifestations (e.g., lung involvement) (143). Due to various mediators involved in the pathogenesis, a whole range of potential novel therapeutic targets are available.

Despite the fact that there are growing everyday clinical experiences with the off-label use of IVIG in idiopathic inflammatory myopathies (IIMs), the results of its effect in dermatomyositis (DM) patients in a phase 3 trial only came out recently (48). Here, the every 4 week use of IVIG at a 2 g/kg dose was compared to placebo for 16 weeks, followed by an open-label extension phase for another 24 weeks (48). While IVIG was superior to placebo according to the Total Improvement Score, the therapy caused more thromboembolic events (48).

Apremilast is a selective inhibitor of the phosphodiesterase-4 (PDE-4) enzyme. In a phase 2a clinical trial, apremilast was tested as an add-on treatment in eight dermatomyositis patients (49). In this study, apremilast was well-tolerated, and there were no severe adverse events, while the overall response rate (ORR) was 87.5%, and the decrease of the cutaneous disease activity severity index (CDASI) score was significant after 3 months of treatment (49). These results raise the possibility that investigating the potential therapeutic benefit of apremilast in an extended clinical study is reasonable.

Increased heat shock protein (HSP) production as a response to toxic cellular changes is often not sufficient in patients with inclusion body myositis (IBM). In recent years, the “heat shock response” amplifier, arimoclomol, was the candidate drug of two phase 2 clinical trials. In the first clinical trial, a slower decline was observed in almost all physical function and muscle strength parameters in the arimoclomol group (50). However, in the second study, arimoclomol did not show beneficial effects compared to placebo (144). Despite these controversial results, the effect of arimoclomol is being tested in patients with IBM in a phase 3 trial (ClinicalTrials.gov Identifier: NCT04049097).

In a recently published case report, anti-CD19 CAR T cell therapy was used in a patient with refractory antisynthetase syndrome (145). After the treatment, transient myalgia appeared, and an increased creatinine kinase level was detected; however, shortly after, an improvement was observed in the physical function parameters (145). In parallel with this, the activity of the disease-associated interstitial lung disease also greatly declined with a massive drop in the serum anti-Jo-1 titer (145). (It is important to emphasize that since CAR T cell therapy showed efficacy on a small group of patients with SLE, one SSc patient, and one IIM patient; a phase 1 clinical trial was recently started with increased numbers of SLE, SSc, pSS, and IIM patients (ClinicalTrials.gov Identifier: NCT06056921).

A number of phase 2 or 3 clinical trials with IIM patients are currently ongoing, for example, with tofacitinib (ClinicalTrials.gov Identifier: NCT05400889), baricitinib (ClinicalTrials.gov Identifiers: NCT04972760 and NCT04208464), abatacept (ClinicalTrials.gov Identifier: NCT03215927), or rituximab (146). In the latter study, rituximab was not superior to cyclophosphamide in the treatment of ILD, while both therapies increased FVC by week 24; however, rituximab caused fewer adverse events (146).

## Discussion and concluding remarks

Despite several available therapies, some systemic autoimmune diseases are still difficult to treat in general (e.g., Sjögren’s syndrome, systemic sclerosis) or have a significant proportion of patients, where refractory conditions can be considered (e.g., rheumatoid arthritis and SLE). With the help of significant basic, translational, and clinical research, more and more information became available on the pathogenesis of systemic autoimmune diseases. As a consequence, during the last few years, some new therapies have been approved by the FDA and/or by the EMA. These include filgotinib for RA, anifrolumab and voclosporin for SLE, or avacopan for GPA and microscopic polyangiitis (MPA). Meanwhile, intensive research also contributed to the development and testing of several promising new candidates, which may cause smaller or bigger revolutions in the management of these diseases. It is important to note that study design is crucial to gain relevant information out of clinical trials; it is especially true for autoimmune connective tissue diseases (e.g., SLE), where some off-label therapies (e.g., rituximab) seem to work in selected cases in the everyday clinical routine but failed to show significant differences over placebo in clinical trials (56). Another important issue is safety: several drugs in clinical studies have massive immunosuppressive effects; in addition to showing beneficial features in the treatment, some therapies may have harmful side effects that need to be closely followed.

Despite the fact that there are many differences in the pathogenesis of systemic autoimmune diseases, many aspects are similar, leading to common molecular targets, like type I IFN/IFN receptor, IL-17, GM-CSF receptor, BAFF, CD40/40L, or JAK/STAT (**Table 2**). If only a quarter of the reviewed drugs targeting these molecules appear on the market and can be used in the therapy of systemic autoimmune diseases, a better new era will be guaranteed. The appearance of novel therapeutic options will surely cause several comparison studies and will lead to newer treatment recommendation guidelines. Well-working and approved novel therapeutic agents may also help to identify new disease subtypes and might potentiate the development of more drugs with similar modes of action (causing a positive feedback loop in drug development in this field). However, we should not forget that optimism cannot substitute carefulness, and the new therapies should be handled with care.

**Table 2:** Common drug targets and similarities.

Target	SLE	RA	pSS	SSc	LVV	AAV	IIM
Type I IFN/IFNR	**✓**		**✓**	**✓**			
IL-17/IL-17R				**✓**	**✓**		
GM-CSFR		**✓**			**✓**		
BAFF/BAFF receptor	**✓**		**✓**	**✓**			
CD40/40L	**✓**	**✓**	**✓**				
JAK/STAT	**✓**	**✓**	**✓**	**✓**	**✓**	**✓**	**✓**
IL-6/IL-6R		**✓**		**✓**			
CD20	**✓**	**✓**	**✓**	**✓**	**✓**	**✓**	**✓**
CD19	**✓**			**✓**			**✓**
CD38	**✓**					**✓**	

AAV, ANCA-associated vasculitis; GM-CSFR, GM-CSF receptor; IFNR, interferon receptor; IL-6R, IL-6 receptor; IL17R, IL-17 receptor; IIM, idiopathic inflammatory myopathy; LVV, large-vessel vasculitis; pSS, primary Sjögren’s syndrome; RA, rheumatoid arthritis; SLE, systemic lupus erythematosus; SSc, systemic sclerosis.

## Author contributions

All authors listed have made a substantial, direct, and intellectual contribution to the work and approved it for publication.
